# 

**Published:** 1869-01-01

**Authors:** 


### THE CHICAGO MEDICAL JOURNAL.

*PUBLISHED SEMI-MONTHLY.*

J. Adams Allen, M.D., LL.D.
EDITOR.
Walter Hay, A.M., M.D.
ASSOCIATE EDITOR.

JANUARY 1, 1869.

CHICAGO:

Church, Goodma[illegible] and Donnelley, Steam Book a[illegible]

108 and 110 Dearborn Street.

